# Precision Medicine for Chronic Diseases: Focus on Lifestyle Changes

**DOI:** 10.3390/jpm12081291

**Published:** 2022-08-05

**Authors:** Domenico Di Raimondo

**Affiliations:** Department of Health Promotion, Mother and Child Care, Internal Medicine and Medical Specialties (ProMISE) “G. D’Alessandro”, University of Palermo, Piazza delle Cliniche, 90127 Palermo, Italy; domenico.diraimondo@unipa.it; Tel.: +39-091-6552180

Virtually all chronic diseases are sustained by lifestyle factors. Poor dietary habits, sedentary living, cigarette smoking, high levels of stress, an unsatisfactory sleep duration and quality, and many other less important factors are driving the rapid and progressive increase in the prevalence of various chronic diseases, including cardiovascular disease, dysmetabolic disorders, several forms of cancer, and chronic obstructive lung diseases but also immune dysfunction and neurodegenerative disorders. Even in counties where—until a few decades ago—rural habits were maintained, the rapidly increasing rate of globalization has expanded the number of individuals that have been impacted; nevertheless, the numerous major epidemiological, cultural, religious, and historical differences between countries render a comprehensive approach to lifestyle changes extremely challenging. Despite major public health efforts aimed at adopting and maintaining a healthy lifestyle, copious epidemiological data indicate that the results obtained are largely unsatisfactory, especially with regard to healthy eating and physical activity, with most of the population far from meeting the current recommendations. Therefore, diet and exercise deserve special attention, as they represent the main targets of intervention and research.

The many research studies that have attempted to clarify the benefits provided by maintaining an adequate level of physical activity (and the harm caused by sedentariness) have highlighted many extremely interesting points that have been the basis for the current recommendations given to both the general population and to specific categories of patients suffering from particular chronic diseases. Since resistance and endurance training lead to the activation of different signaling pathways and result in distinct adaptations of muscles and target organs, not all types of exercise fit all people. Targeted exercise programs should be used for the prevention and/or the treatment of various chronic diseases. The training intensity and the optimal duration of each session of exercise and of the entire training program must also be individualized [1]. Much has been written but even more remains yet to be investigated. The study of the mechanisms by which regular exercise can influence the metabolism of tissues, organs, and systems far from the contracted muscle, and thus preserving an optimal healthy state for the entire body, is an extremely attractive field of research. Since the first report dating back to 1991, thousands of papers concerning several different peptides found as mRNA expressed by skeletal muscle tissue or released in the circulation during prolonged muscular contraction have been published. These peptides have been labeled as putative “muscle-derived factors”, “muscular cytokines or myokines”, “exercise factors”, or “muscular growth factors” [2]. The rapid accumulation of such a large amount of data about the muscle secretome has led to the parallel increase in questions about the true role played by the secretory activity of the muscle in determining the chronic adaptation to regular exercise and the reduction in the risk of mortality and morbidity in the trained subjects [3]. This is a fascinating area of research that we have only recently begun to address.

If the goal to combat inactivity and to increase the awareness and emphasis on an active lifestyle is not biased (or at any rate, is only limitedly affected) by the country in which the action is taken, the same cannot be said about diet. Dietary habits differ greatly between peoples and age groups, and depend on religion, work habits, food availability, traditions, and culture. Determining how healthy a specific diet (or food) may be is not always easy. Virtually every single nation provides its own recommendations, and although the Mediterranean diet is regarded as a benchmark to be followed [4,5,6,7,8], many aspects of its potential benefits have yet to be clarified.

Moreover, in recent decades, in an attempt to fight the growing prevalence of obesity and obesity-related dysmetabolic disorders, we have witnessed a multiplication of various new diets, supplements, or new nutritional approaches, whose actual short- and long-term benefits are all yet to be proven [9,10].

The conceivable interventions to prevent the occurrence of chronic diseases in healthy individuals promoting a safer lifestyle are virtually endless, from national and international health policy interventions to the creation of targeted advertising media programs to the development of more effective monitoring and telemedicine systems, to mention just a few. Scientific research plays a central role in providing practitioners—and through them, a larger audience—with reliable and reproducible data on which to determine their interventions. Numerous pieces of evidence are available, but much remains to be implemented and the goal must be high quality research. A very recent review of the available Randomized Controlled Trials (RCTs) addressing the issue of behavioral counseling interventions to promote a healthy diet and physical activity for cardiovascular disease prevention in adults [11] allows us to the highlight the relevant gaps in the literature that must be immediately addressed. The following is just a small example of how much still needs to be discovered/researched on a topic so important to everyone’s health, and it is also meant to be a suggestion for potential contributors to the Special Issue, for which I serve as a Guest Editor:

- We have only scarce data on the effectiveness of specific dietary and/or exercise interventions among different populations;
- We need the standardization of behavioral outcomes;
- An insufficient number of studies have evaluated the comprehensive interventions designed to reduce sedentary behaviors and limited data are available on longer-term health outcomes;
- Limited data are available on the new frontier of wearable activity trackers and on the many opportunities offered by new technologies;
- Only a few studies have investigated the effects of different dietary regimens (ketogenic diets, low carbohydrate diets, paleolithic diets, etc.) undertaken with the aim of improving health the status in specific groups of subjects;
- Limited, low-quality studies have compared the effectiveness of different combined approaches, such as “diet + exercise + counseling”, vs standard approaches;
- Targeted research is needed to address the importance of the social determinants of health (availability, costs, sustainability, etc.), which test different interventions that are able to positively affect access to healthy eating and improved levels of physical activity;
- In a world that will likely have to learn to live with an endemic COVID-19, we have to ascertain the best ways to engage, inform, and periodically reassess patients; discover an optimal method for training physicians and health personnel; and harness the efficacy of teamwork to achieve better results.
